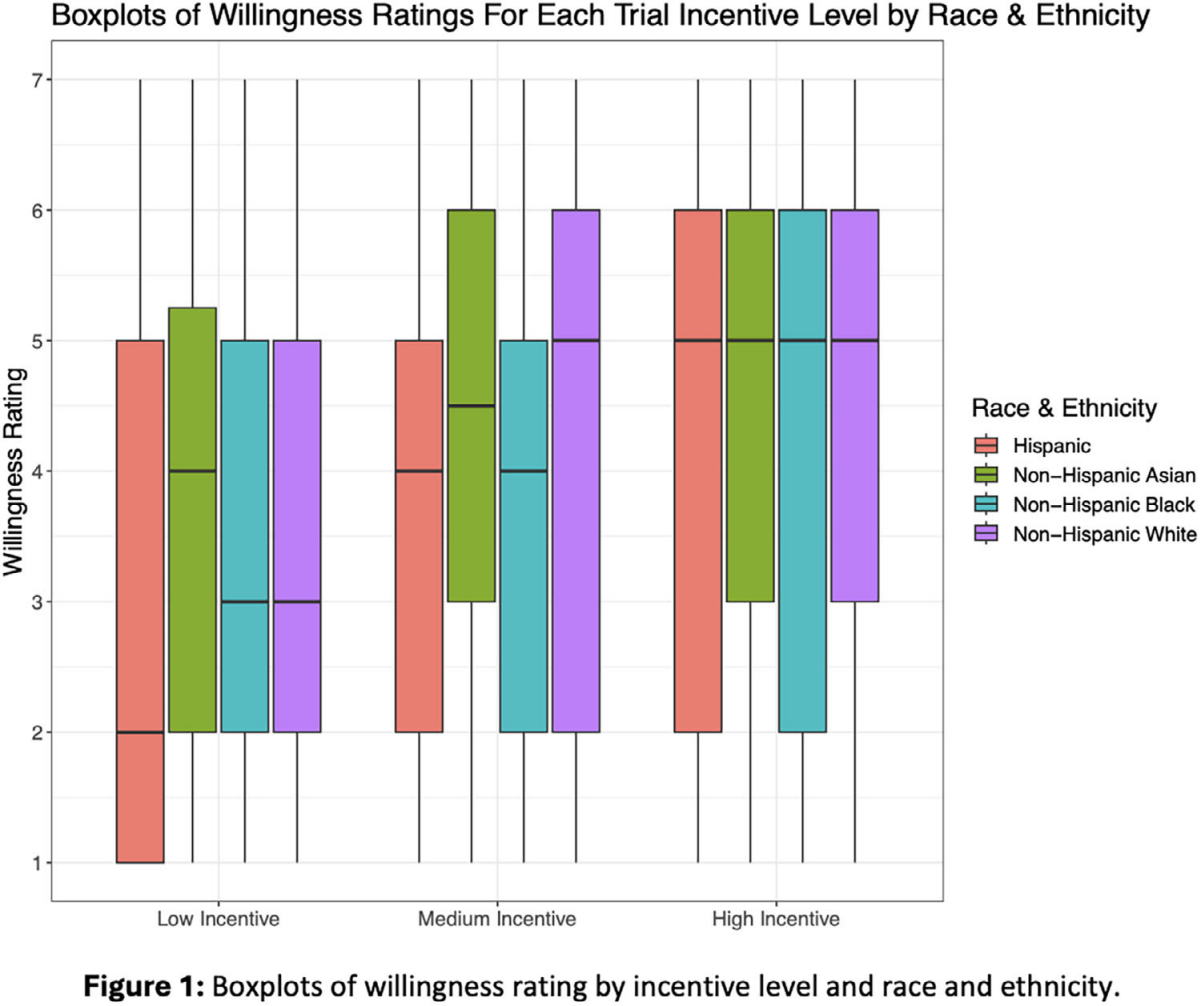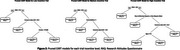# Exploring Characteristics Predictive of Willingness to Participate in Preclinical Alzheimer’s Disease Trials

**DOI:** 10.1002/alz70859_100591

**Published:** 2025-12-25

**Authors:** Adam I Birnbaum, Kristin Harkins, Jason Karlawish, Josh D Grill, Daniel L Gillen

**Affiliations:** ^1^ University of California, Irvine, Irvine, CA USA; ^2^ University of Pennsylvania Perelman School of Medicine, Philadelphia, PA USA; ^3^ The UC Irvine Institute for Memory Impairments and Neurological Disorders, Irvine, CA USA; ^4^ Institute for Memory Impairments and Neurological Disorders, University of California, Irvine, Irvine, CA USA

## Abstract

**Background:**

Recruitment into Alzheimer’s disease (AD) clinical trials remains challenging. Efficiently identifying individuals who are more willing to enroll can inform recruitment strategies.

**Methods:**

Participants were interviewed to assess willingness to participate in preclinical AD trials of various incentive levels: a high‐incentive scenario providing transportation, a lifetime dementia risk assessment, summaries of cognitive testing results, and high financial compensation, a medium‐incentive scenario providing only cognitive results and high financial compensation, and a low‐incentive scenario providing none of these incentives. Participants rated willingness on a 7‐point Likert scale. We assessed willingness across racial and ethnic groups via boxplots. To identify participant characteristics predictive of willingness, we collapsed willingness into a binary construct defining 6‐7 as “willing” and fit classification and regression trees (CARTs). We used random forests to identify differences in feature importance via mean decrease in accuracy across racial and ethnic groups in the high‐incentive scenario.

**Result:**

Among 261 participants, 69 identified as Hispanic, 69 as Non‐Hispanic (NH) White, 45 as NH Black, 72 as NH Asian, 5 as NH other, and 1 did not report race or ethnicity. Figure 1 gives grouped boxplots of willingness stratified by race and ethnicity (NH other omitted due to limited sample size). Willingness was similar across racial and ethnic groups in the high‐incentive scenario. Willingness among Hispanic participants skewed lower than their NH peers in the low‐incentive scenario. Figure 2 gives pruned CARTs for each scenario. Favorable research attitudes, measured by the Research Attitudes Questionnaire (RAQ), and higher levels of concern about AD were predictive of higher willingness in all scenarios. Higher income was predictive of higher willingness in the low‐incentive scenario. The random forest analysis identified research attitudes and concerns about AD as the two most important characteristics in all racial and ethnic groups except among Hispanic participants, where measures of global cognition (Montreal Cognitive Assessment) and subjective cognitive complaints (Cognitive Function Instrument) were the most predictive.

**Conclusion:**

The RAQ and concerns about AD dementia may be low‐cost tools for identifying willing participants. Few differences were observed across racial and ethnic groups. Future work might investigate the relationship between the RAQ and willingness among Hispanic individuals.